# Suboptimal evolutionary novel environments promote singular altered gravity responses of transcriptome during *Drosophila* metamorphosis

**DOI:** 10.1186/1471-2148-13-133

**Published:** 2013-06-27

**Authors:** Raul Herranz, Oliver J Larkin, Richard JA Hill, Irene Lopez-Vidriero, Jack JWA van Loon, F Javier Medina

**Affiliations:** 1 Centro de Investigaciones Biológicas (CSIC), Ramiro de Maeztu 9, E-28040, Madrid, Spain; 2 DESC (Dutch Experiment Support Center)/MMG-Lab, European Space Research & Technology Center – European Space Agency (ESTEC-ESA) Keplerlaan, 1 2201 AZ, Noordwijk, The Netherlands; 3 School of Biosciences, University of Nottingham, Sutton Bonington Campus, Loughborough LE12 5RD, UK; 4 School of Physics & Astronomy, University of Nottingham, Nottingham NG7 2RD, UK; 5 Centro Nacional de Biotecnología (UAM-CSIC), Madrid, Spain; 6 Department Oral and Maxillofacial Surgery/Oral Pathology, VU University Medical Center, Amsterdam, The Netherlands; 7 Department Oral Cell Biology - Academic Centre for Dentistry Amsterdam (ACTA), VU University, Amsterdam, The Netherlands

**Keywords:** Evolutionary genomics, Gene family evolution, Microgravity-hypergravity, Magnetic levitation, Gene expression, Microarray

## Abstract

**Background:**

Previous experiments have shown that the reduced gravity aboard the International Space Station (ISS) causes important alterations in *Drosophila* gene expression. These changes were shown to be intimately linked to environmental space-flight related constraints.

**Results:**

Here, we use an array of different techniques for ground-based simulation of microgravity effects to assess the effect of suboptimal environmental conditions on the gene expression of *Drosophila* in reduced gravity. A global and integrative analysis, using “gene expression dynamics inspector” (GEDI) self-organizing maps, reveals different degrees in the responses of the transcriptome when using different environmental conditions or microgravity/hypergravity simulation devices. Although the genes that are affected are different in each simulation technique, we find that the same gene ontology groups, including at least one large multigene family related with behavior, stress response or organogenesis, are over represented in each case.

**Conclusions:**

These results suggest that the transcriptome as a whole can be finely tuned to gravity force. In optimum environmental conditions, the alteration of gravity has only mild effects on gene expression but when environmental conditions are far from optimal, the gene expression must be tuned greatly and effects become more robust, probably linked to the lack of experience of organisms exposed to evolutionary novel environments such as a gravitational free one.

## Background

Genome-wide transcriptional profiling in flies exposed to a reduced gravity level in the International Space Station (ISS), or in simulated microgravity (using a Random Positioning Machine, RPM), is severely altered [1]. These important alterations in *Drosophila* gene expression are intimately linked to imposed spaceflight-related environmental constraints (i.e. uncontrollable temperature during transport, launch and travel to the ISS plus other spaceflight hardware container constraints, such as limited amount of oxygen, light or humidity supply) during *Drosophila* metamorphosis; the alterations do not appear when similar experiments are performed in optimal environmental conditions [1].

Two experimental approaches can be used to evaluate the effects of altered gravity. The first of these is to perform experiments in orbit, where the *g* force is reduced by three orders of magnitude compared to the force of gravity on the ground. However, access to spaceflight opportunities is problematic, expensive and scientifically constrained. The second approach is to use a Ground Based Facility (GBF) which balances the force of gravity, or otherwise neutralizes the effects of gravity on the organism [2,3]. The effects of Earth’s surface gravity on an organism can be lessened or neutralized by means of a mechanical device that constantly changes the direction of the effective *g* force (g*) with respect to the sample, i.e. using a classical horizontal 2D clinostat or a random positioning machine (RPM); the latter is a 3D version of the clinostat that continuously randomizes the orientation and speed of rotation [4,5]. To enhance our understanding of altered gravity effects, we also employ mechanical simulation of a hypergravity environment, *i.e.*, a centrifuge with a large enough radius that shear forces in the sample chamber are reduced to an acceptable level [6,7]. An example of such a GBF is the Large Diameter Centrifuge (LDC) located at ESA research center in The Netherlands (ESTEC) [8].

A different kind of reduced gravity simulator, free of the rotational, mechanical and inertial forces generated by spinning simulators and with the advantage of acting at the molecular level, is based on diamagnetic levitation [9-13]. Diamagnetic material, such as water, is repelled from a magnetic field. Since the composition of the majority of biological tissues is largely water, this technique can also levitate living organisms (0g*), with exposures to hypergravity (2g*) and magnetic field control conditions (1g*) simultaneously and in the same environment [14].

Here, we study the effects of altered gravity conditions on the gene expression profile of *Drosophila melanogaster* during metamorphosis (3–4 day-long experiments), using three GBFs and whole genome microarray platforms. In addition, we study the effect of applying two environmental constraints to the system, a cold step of three days at 12°C (ΔT) and a containment of the samples in a chamber that reduces the amount of available oxygen (↓O_2_), both of them not reaching deleterious doses [15,16]. Both environmental parameters were found to be crucial in previous studies in real and simulated space environments [1] and in a preliminary attempt at using magnetic levitation as a microgravity simulator with *Drosophila*[17]. The main and novel conclusion reported here is that *Drosophila* responses to altered gravity environments are variable, being greatly dependent on environmental conditions and the type of GBF used, despite some common stress response and behavioral effects confirmed by altered expression of related genes.

## Methods

### Ground based facilities

Two mechanical (at DESC/ESTEC in Noordwijk, The Netherlands) and one magnetic (at the University of Nottingham in the United Kingdom) GBFs have been used to generate altered gravity conditions. In the first set of experiments, microgravity was simulated mechanically, using a random positioning machine (RPM) [4,5] and hypergravity conditions were applied with a large centrifuge (LDC) [8] in the same location and simultaneously. The samples were exposed in similar type I containers used in a former experiment in the ISS [1].

In the second set of experiments, a high gradient superconducting magnet in simulated microgravity and hypergravity samples within the bore of the magnet; the forces acting on the samples depend on the position of the object in the magnet bore. This facility is not compatible with a type I metallic container, so experiments were performed in a 25 mm-diameter, 10 mm-tall ‘arena,’ constructed within a 25 ml clear plastic sample tube, positioned near the top of the bore, 80 mm above the centre of the solenoid. Here, the magnitude of the field *B* and its vertical field gradient ∂*B*/∂*z* is large enough to levitate biological tissue. At the centre of the arena, where the product *B* times ∂*B*/∂*z* is 1360 T^2^/m, the diamagnetic and gravitational forces on water balance exactly. We label this point the ‘0g* point’, and correspondingly refer to the arena enclosing it as the ‘0g* arena’. The magnitude of the effective gravity acting on water increases away from the 0g* point, but is within a few percent of *g* everywhere inside the 0g* arena. The asterisk on the label ‘0g*’ is used as a reminder that the effective gravity refers to water, and that there is a strong magnetic field present (11.5 tesla). An arena located at the centre of the bore, where there is no vertical gradient of the field and hence no vertical force balancing gravity, is used to control for additional effects of the magnetic field besides that of levitation: here the effective gravity is unchanged. We label this arena ‘1g*’. The magnetic field in this position is 16.5 tesla. In the lower region of the solenoid, 80 mm below the centre of the solenoid, a third arena labeled ‘2g*’ was placed where the magnitude of the effective gravity within the arena was within a few percent of 2*g* (hypergravity simulated position) and directed downwards [9-12]. In summary, experiments were done on three samples in the magnet (11.5 tesla at the 0g* and 2g* points, and 16.5 tesla at the 1g* point) to simulate both microgravity by levitation (0g*) and simulated hypergravity (2g*) and a 1*g* external control was performed simultaneously in all GBFs, placed well away from the magnet in this GBF.

### Biological materials

We used a similar experimental procedure to that used in a previous ISS experiment [1] developed during the 14-days Cervantes Mission to the ISS on October 2003, in all three GBFs. Late instar larvae of *Drosophila melanogaster* Oregon R, just before entering metamorphosis, were collected and placed on filter paper, and then exposed to altered gravity for 4 days at 22°C until just before the end of metamorphosis. Two types of environmental perturbations were added: one is a 3.5 days cold step at 14°C prior to the experiment (ΔT) and the other is to hold the samples in the same hermetically sealed type-I container used in space experiments ([1], limited amount of oxygen available, ↓O_2_, versus samples in open containers (air permeable membranes)). To analyze the transcriptional profile of *Drosophila*, two to four biological replicas, including 8 to 12 individuals from each condition, that passed quantity (nanodrop absorbance measurement) and quality (RNA integrity determined by bioanalyzer) tests of the extracted RNA, were used in microarray analysis. A summary of the conditions and samples used is included in Table 1. All samples were processed using Affymetrix *Drosophila* (DrosophilaGenome2) chips covering nearly the whole fly coding genome. A validation of this approach, including qRT-PCR has been published earlier [1,17].

**Table 1:** **Description of the 90 microarray CEL files used (accession number GEO NCBI database *GSE33779*)**

**Simulation facility**	**Temperature constraints (ΔT)**	**Oxygen (↓O**_**2**_**) constraints**	**g level**	**Name of CEL file replicates**			
RPM (From early to late pupae just before imagoes hatching)	No	No (open)	Sim μ*g*^	80A	80B	80C	
			1*g*	81A	81B	81C	
	Yes	No (open)	Sim μ*g*^	70A	70B	70C	
			1*g*	71A	71B	71C	
	Yes	Yes (close)	Sim μ*g*^	70E	70F	70G	70H
			1*g*	71E	71F	71G	71H
Magnetic levitator (From early to late pupae just before imagoes hatching)	No	Yes (close)	0g*	60A	60B	60C	
			1g*	61A	61B	61C	
			2g*	62A	62B	62C	
			1*g*	6cA	6cB	6cC	
			1*g* (open)	6oA	6oB	6oC	
	Yes	Yes (close)	0g*	50A	50B	50C	
			1g*	51A	51B	51C	
			2g*	52A	52B	52C	
			1*g*	5cA	5cB	5cC	
			1*g* (open)	5oA	5oB	5oC	
Hypergravity centrifuge (6*g*/12*g*) (From early to late pupae just before imagoes hatching)	No	No (open)	12*g*^	Q4A	Q4B	---	
			6*g*^	---	Q3H^#^	Q3I^#^	
			1*g*^	Q0A	Q0H^#^	Q0I^#^	
			1*g*	Q1A	Q1H^#^	Q1I^#^	
	Yes	No (open)	12*g*^	R4A	R4B	R4C	
			6*g*^	---	R3H^#^	R3I^#^	
			1*g*^	R0A	R0H^#^	R0I^#^	
			1*g*	R1A	R1H^#^	R1I^#^	
	No	Yes (close)	12*g*^	Q9A^#^	Q9B^#^	Q9C^#^	
			6*g*^	---	Q8H^#^	Q8I^#^	
			1*g*^	QCA	QCB	QCC	
			1*g*	Q6A	Q6B	Q6C	
	Yes	Yes (close)	12*g*^	---	Q9E^#^	Q9F^#^	
			6*g*^	---	R8H^#^	R8I^#^	
			1*g*^	RCA	RCB	---	
			1*g*	R6A	R6B	---	

Comparison experiments were performed using different environmental constraints in three devices. ΔT constraints mean 3.5 days of cold step (12°C) then 4 days at 22°C following the GENE experiment temperature profile [1]. ↓O_2_ means oxygen amount limited by the container (closed). * indicates the presence of a high magnetic field; ^ indicates the presence of mechanical/rotational disturbances; ^#^ indicates that replicates have been performed in a parallel second experiment with the same design. The name assigned to any of CEL file replicates is provided for clarity when revisiting the dataset.

### Microarray data analysis

After data processing as indicated in [17], each probe was tested over replicates for changes in expression between different conditions using an empirical Bayes moderated t statistic, *i.e.* Limma [18]. To control the false discovery rate (FDR), p values were corrected using the method of Benjamini and Hochberg [19]. FIESTA viewer (http://bioinfogp.cnb.csic.es/tools/FIESTA/index.php) was used to visualize all microarray results and to evaluate the numerical thresholds applied for selecting differentially expressed genes [20]. Probe set lists were filtered using raw limma or FDR p-values from the FIESTA viewer interface. This whole-genome expression data has been submitted to GEO NCBI database with the accession number GSE33779.

### Gene ontology and whole genome GEDI analysis

Gene ontology was analyzed in the selected probe set lists by DAVID GO functional annotation clustering [21,23]. Only gene ontology clusters that appear recurrently through the examination of the different lists have been considered in the analysis of Tables 2 and 3. A global and integrative analysis using “gene expression dynamics inspector” (GEDI) self-organizing maps, was performed using the above indicated software v2.1 [24]. Using transformed and corrected signal log2ratios data, we identified 11594 probe sets that show signal log2ratio changes > 0.5 or < −0.5 relative to the 1*g* control in at least one of the experimental conditions. Mosaics of 20 × 15 grid size (average of 39 probe sets/tile) were obtained using the self-organizing maps algorithm and standard settings of the software [24] using the signal log2ratio of the selected probesets. The average signal log2ratio for each tile or cluster of probesets was calculated and displayed in panels for all of the experimental conditions analyzed and also for rotational controls in the LDC and ↓O_2_ condition controls for the magnet.

**Table 2:** DAVID gene ontology functional annotation enrichment analysis in mechanical simulators

**GBF**	**ΔT**	↓**O**_**2**_	**G level**	**Affected genes (limma raw(FDR))**	**Behaviour responses**		**Metamorphosis/morphogenesis/organogenesis**	**Stress responses**		**GO clusters observed also in control conditions**				
					**Reproductive/ mating/oviposition**	**Sensorial/ hormonal/ odorant prot.**		**Abiotic (heat, light & hypoxia)**	**Biotic (defense & immune sys.)**	**Insect cuticle proteins**	**Cytoesqueleton and cell adhesion**	**Proteolysis**	**Red-Ox states/energy**	**Hemo/ metals/ions binding**
RPM	No	No	sim μ*g* UP	218(0)		*2.9×10*^*-4*^						3.5×10^-2^	*2.5×10*^*-6*^	
			sim μ*g* Down	117(0)			**8.7**×**10**^**-9**^				2×10^-2^			3.9×10^-3^
	Yes	No	sim μ*g* UP	28(0)		1.1×10^-3^								
			sim μ*g* Down	17(0)						1.9×10^-3^				
	Yes	Yes	sim μ*g* UP	20(0)										
			sim μ*g* Down	21(0)										
LDC	No	No	12*g*^ UP	85(0)								7×10^-2^	**<10**^**-14**^	4.2×10^-3^
			12*g*^ Down	63(0)			1.7×10^-3^			1×10^-3^				
			6*g*^ UP	37(0)			*2.4×10*^*-5*^							1.1×10^-2^
			6*g*^ Down	8(0)					1×10^-2^					
	Yes	No	12*g*^ UP	873(185)	**1.5**×**10**^**-16**^	7.9×10^-2^	*3×10*^*-4*^		**1.9**×**10**^**-8**^					
			12*g*^ Down	614(72)						**4.1**×**10**^**-12**^	*8.9×10*^*-4*^		*5.8×10*^*-5*^	5.7×10^-3^
			6*g*^ UP	288(0)				3.9×10^-2^					*2*×*10*^*-5*^	3.7×10^-2^
			6*g*^ Down	118(0)			2.9×10^-3^	*2.9×10*^*-5*^	2.3×10^-2^		1.7×10^-2^			3.5×10^-2^
	No	Yes	12*g*^ UP	>10^3^(1557)	**4.1**×**10**^**-10**^		**5.9**×**10**^**-15**^				*1.2×10*^*-4*^			
			12*g*^ Down	>10^3^(1040)				4.7×10^-3^		**8.4**×**10**^**-22**^			**5.1**×**10**^**-7**^	3.4×10^-3^
			6*g*^ UP	>10^3^(1359)	1.3×10^-3^		**<10**^**-15**^		7×10^-2^	*3×10*^*-6*^	**1.5**×**10**^**-16**^			*5.3×10*^*-4*^
			6*g*^ Down	912(720)	*7.4*×*10*^*-4*^	*6.8*×*10*^*-5*^		*3.1×10*^*-6*^	1.7×10^-2^			**1.9**×**10**^**-9**^	**5.4**×**10**^**-11**^	**5.8**×**10**^**-7**^
	Yes	Yes	12*g*^ UP	608(6)	**9**×**10**^**-11**^		**3**×**10**^**-14**^	*5.1×10*^*-4*^	3.7×10^-3^		**2.8**×**10**^**-11**^			**3.9**×**10**^**-7**^
			12*g* Down	334(0)		*5.1×10*^*-4*^							*9.7×10*^*-5*^	
			6*g* UP	419(1256)	**4.9**×**10**^**-17**^	6.1×10^-2^	3×10^-2^		7.8×10^-2^		3.7×10^-2^	8×10^-2^	*7×10*^*-6*^	
			6*g* Down	297(912)				**9.6**×**10**^**-12**^			*2.5×10*^*-4*^		**7.7**×**10**^**-8**^	*7×10*^*-4*^

The number of up- or down-regulated probesets in each GBF facility (first column) and condition (column 2 to 4) has been calculated and is shown in the fifth column (filtering with a p-value limma < 0.001, except for the LDC conditions (p-value FDR < 0.05 due to the large number of probesets detected)). Relative enrichment analysis of these gene lists have been done, and the more frequent GO groups observed in experimental (columns 6 to 10), or 1*g*/environmental controls (columns 11 to 15) are included. Statistically meaningful p values are indicated in the table using italics and bold fonts to emphasize a greater level of enrichment in each particular GO group is observed. ^ indicates the presence of mechanical/rotational disturbances.

**Table 3:** DAVID gene ontology functional annotation enrichment analysis in magnetic simulators

**GBF**	**ΔT**	↓**O**_**2**_	**G level**	**Affected genes (limma raw(FDR))**	**Behaviour responses**		**Metamorphosis/morphogenesis/organogenesis**	**Stress responses**		**GO clusters observed also in control conditions**				
					**Reproductive/ mating/oviposition**	**Sensorial/ hormonal/ odorant prot.**		**Abiotic (heat, light & hypoxia)**	**Biotic (defense & immune sys.)**	**Insect cuticle proteins**	**Cytoesqueleton and cell adhesion**	**Proteolysis**	**Red-Ox states/energy**	**Hemo/ metals/ions binding**
Mag	No	Yes	0g* UP	61(0)			4.5×10^-2^				3.8×10^-2^			
			0g* Down	62(0)	**1.7**×**10**^**-19**^							*6.2×10*^*-4*^		
			1g* UP	17(0)										
			1g* Down	25(0)								3.4×10^-2^		
			2g* UP	420(30)	6.4×10^-2^	8.8×10^-3^	*2.1×10*^*-4*^	1.3×10^-2^	1×10^-2^	**5**×**10**^**-8**^	*6.7×10*^*-4*^			3.1×10^-3^
			2g* Down	328(42)	**6.1**×**10**^**-22**^	*1.2×10*^*-5*^			6.6×10^-2^			*4.4×10*^*-6*^	**2.9**×**10**^**-8**^	
	Yes	Yes	0g* UP	635(458)	*1.9×10*^*-4*^	*7.7×10*^*-5*^		1.9×10^-3^	2.7×10^-2^			*1.1×10*^*-5*^	**4.2**×**10**^**-12**^	*1.7×10*^*-6*^
			0g* Down	524(349)	2×10^-2^	2.3×10^-3^		3.4×10^-2^	1.5×10^-2^	**2**×**10**^**-25**^		2.9×10^-3^	**7.2**×**10**^**-11**^	*4.7×10*^*-5*^
			1g* UP	350(196)		2.9×10^-3^		1×10^-3^	1.4×10^-3^			1.1×10^-2^	**1.1**×**10**^**-11**^	**2.9**×**10**^**-8**^
			1g* Down	191(97)						**2.5**×**10**^**-18**^		2.7×10^-2^		
			2g* UP	208(7)					1.4×10^-2^				1.2×10^-6^	*8.6×10*^*-5*^
			2g* Down	157(2)						**2.3**×**10**^**-15**^		6.8×10^-2^		

The number of up- or down-regulated probesets in each GBF facility (first column) and condition (column 2 to 4) has been calculated and is shown in the fifth column (filtering with a p-value limma < 0.001). Relative enrichment analysis of these gene lists have been done, and the more frequent GO groups observed in experimental (columns 6 to 10), or 1*g*/environmental controls (columns 11 to 15) are included. Statistically meaningful p values are indicated in the table using italics and bold fonts to emphasize a greater level of enrichment in each particular GO group is observed. * indicates the presence of a high magnetic field.

## Results

### Simulated microgravity/hypergravity produce greater effects in the overall gene expression pattern during *Drosophila* metamorphosis when in combination with suboptimal environmental parameters

The gene expression profile was evaluated in *Drosophila* pupaes exposed to altered gravity during the whole metamorphosis stage. Two hypergravity and two microgravity simulators were used and up to four environmental conditions applied (combinations with or without a three day-long cold step at 12°C before simulation (ΔT) and oxygen limitation (↓O_2_) environments). The number of probesets that show a statistically meaningful (using raw or FDR corrected limma p-values) signal log2 expression ratio in each condition is shown in Tables 2 and 3 (see Additional file 1 for affected genes lists).

The analysis of the results from the RPM microgravity simulator (Table 2) shows a very low number of genetic alterations when using the limma p-value algorithm (this number becomes zero if we apply the stringent FDR correction). This indicates that this simulator has very little effect, even in the case of the most severely constrained environment (ΔT/↓O_2_). In the case of magnetic levitation (Table 3), (0g* position in the magnet) we observe less than a hundred variations, except in the ΔT/↓O_2_ environment, in which the number of probesets with altered expression increases to 635/524 up/down-regulated genes. The hypergravity position (2g*) in the magnet causes a larger number of variations than the internal magnet control sample (1g*, exposed to magnetic field but not to altered gravity). Effects of the magnetic field (i.e. other effects of the strong magnetic field, in addition to the levitation force) are obvious in the double-constrained environment (ΔT/↓O_2_), especially if we apply the FDR correction algorithm.

The LDC experiments (Table 2) show the clearest increase in the number of affected genes in relation to the introduction of suboptimal environmental conditions. Under optimal environmental conditions we find almost no effects. Some effects, similar to the ones observed in the magnet, appear under ΔT conditions, but using FDR correction, the differences only appear in the 12*g* condition. Under a hypoxia environment, (both ↓O_2_ and ΔT/↓O_2_) thousands of genes are observed to be affected, even when applying the stringent FDR correction.

### GO groups affected are similar in all experiments

Considering these differences in the number of affected genes in each environmental/facility condition, we decided to analyze what kind of genes are affected by means of DAVID gene ontology enrichment tool [21-23]. Tables 2 and 3 summarize the more frequent GO groups affected, discriminating the ones affected in altered gravity conditions from the ones affected in all samples (both experiments and controls (rotational, magnetic or temperature controls)). In the case of the generally affected clusters, we observed several GO groups related to mechanical and/or energetic stress. Some of them, for instance insect cuticle proteins, are more frequent in down regulated genes. On the other hand, three groups of genes are affected specifically in altered gravity conditions: behavior-related genes (specially related with sexual behavior), external stress responses (both abiotic and biotic responses to external agents) and metamorphosis or organogenesis genes. An example of the genes included in some of these GO clusters has been presented as Figures S1 to S7 (see Additional file 2).

### A genome scale response to altered gravity is more easily detectable under suboptimal environmental conditions

**Figure 1:**
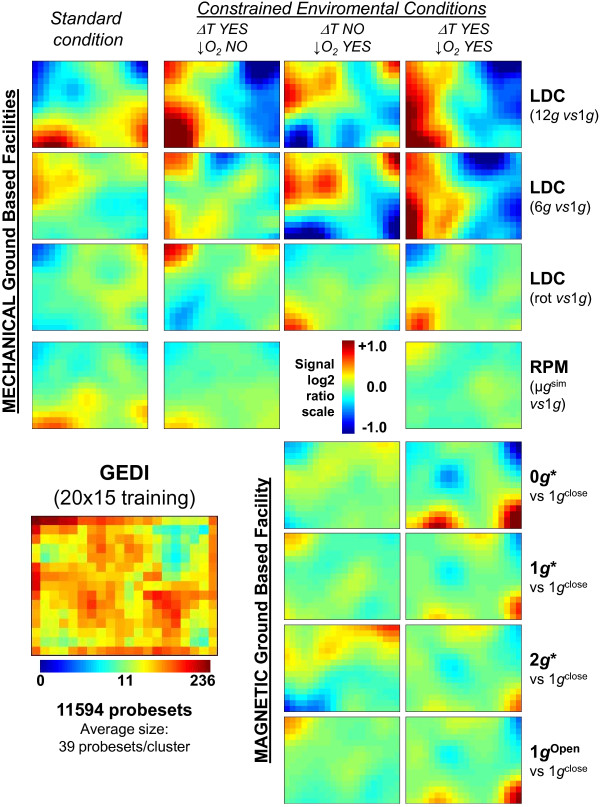
**GEDI 15 × 13 clustering analysis based on the six altered gravity experiments.** Colors in the main panels indicate the average log2ratios level of each cluster in this condition compared to 1*g* controls, following the vertical scale. The lower-left panel indicates cluster density (number of probesets per cluster) following the horizontal scale at the bottom. Comparison experiments in standard cultivation conditions in the lab are shown in the first column (constant temperature and open atmosphere, RPM/LDC). RPM/LDC transcriptome status under different environmental conditions have been shown in the second (ΔT *i.e.*, cold step and open atmosphere), the third (optimal temperature but ↓O_2_, *i.e.* closed container nearly induced hypoxia) and the fourth column (double constrained experiment, ΔT and ↓O_2_). In the case of the magnet facilities, the experiments were performed in closed containers (↓O_2_). Each row represents an altered gravity level or an internal control (12*g*, 6*g*, rotational control, μ*g*^*sim*^ (RPM), 0g*, 1g*, 2g*, 1*g*^*Open*^ container control). * effective gravity is that of pure water (pupae were not levitating in free space, but attached to filter paper).

In order to obtain a global vision of the transcriptome response to the different treatments, we analyzed the microarray data using the “Gene Expression Dynamics Inspector” (GEDI) program [24]. GEDI is a “Self Organizing Map” (SOM) based software that allows the visualization of gene expression patterns in mosaics of *n* x *m* tiles. Each tile corresponds to a cluster of genes that behaves similarly across conditions (centroid). Different colors reflect the expression intensity of a centroid in each condition (in our case the average log2 ratio of intensities compared to 1*g* controls). Additionally, GEDI places similar centroids close to each other in the mosaic, creating an image of the transcriptome and allowing its analysis as an entity by simple visualization and through different conditions. We found that 11594 probe-sets, the ones with significant (raw limma p-value <0,05) expression change in at least one of the comparisons (see Additional file 3 for gene expression values), were clustered by GEDI analysis and were placed in 20 × 15 mosaics with an average of 39 genes per centroid (Figure 1, see Additional file 4 for GEDI analysis file).

The transcriptome shows variable responses to altered gravity depending on the gravity level and the environmental constraints. Paying attention to the log2 expression change ratio scale in the bottom-left corner of Figure 1 we notice that the changes in expression that we are able to discriminate is up to 1 fold for increased or decreased probesets. Consistently with the numbers of differentially expressed probesets in the previous section, the gene expression change is greater when we apply more constrained conditions, but the precise clusters of genes being affected are not the same in the same facility (each row in Figure 1). In contrast, when we compare the two centrifuge experiments (12*g* and 6*g*, first and second row in Figure 1) we observe that the same hypergravity signature is observed depending on the environmental condition.

Only LDC gene expression variations were clearly observed using this visual tool. The changes observed in the magnetic levitation/hypergravity positions or in the RPM are similar to the ones observed in the LDC rotational control, Magnet 1g* internal control or in the open (unconstrained amount of oxygen) magnet external control.

## Discussion

It has been widely observed that *Drosophila* imagoes suffer a marked enhancement in motility partly due to the disorientation of the flies when they are exposed to altered gravity conditions [13,25]. It has been also established that differences in age and gender of the imagoes can influence the degree of responsiveness to microgravity [17,26]. In addition, both real space conditions [27,28] and hypergravity [29,30] have a deep impact on the ageing process of the flies. In our study, due to the sessile nature of *Drosophila* pupae during metamorphosis, behavior alterations cannot be the reason for the observed changes in gene expression, and consequently the number of variations in the simulated microgravity samples is quite low in our samples. In contrast, the differential GO group enrichment (Table 2) points to the fact that some behavior and stress response (biotic and abiotic) genes change their expression when *Drosophila* experiences microgravity/hypergravity. This indicates that the behavior of the imagoes can be influenced by prior exposure to altered gravity exposure during their metamorphosis stage, distinct from behavioral changes brought about by exposure of the adult flies to altered gravity.

A possible explanation for these results relies on the ancestral evolutionary origin of the adaption of living organisms to our Earth gravity. Gravity has been a constant force during evolution. Most animals are able to sense the gravity vector in order to establish their spatial references or to find nutrients, but the genome does not have a particular collection of genes evolved to respond to an altered gravity environment. Thus, the transcriptome has to choose different responses depending on the different environmental parameters that could be present in a certain moment, and additionally it will use particular kinds of gene products, the ones coming from large gene families. All gene clusters included in Table 2 (and detailed in supplementary materials) include at least one large gene family. The main players in any of the five gene clusters included in Table 2 are accessory gland proteins (Affymetrix 2.0 *Drosophila* array contains at least 16 members of the Acp gene family), odorant/pheromone binding proteins or receptors (47 Obp, 62 Or and 5 Pbprp gene family members), ecdysone inducible genes or proteins (11 Eig and 13 Eip members), heat-shock proteins (23 Hsp related gene members) and Turandot/Inmune induced proteins (7 Tot and 7 IM members). In fact, other gene clusters including large gene families have been identified in altered gravity conditions as well as in controls: insect cuticle proteins (more than 70 Cpr plus 13 Lcp (larval) members organized in 9 clusters (Ccp), see [31]), energy/Red-Ox related genes like cytochromes (86 Cyp gene family members), even an unknown function gene family Osiris (20 Osi gene family members, see [32]). It is intriguing that the internal controls used in these GBF experiments also reveal some effects on the gene expression profile. Mechanical- or magnetically-induced stress experienced during simulation may constitute a stimulus similar to the change in the gravity vector itself.

## Conclusions

We have confirmed here the impact of constrained environmental parameters on the response of the transcriptome to altered gravity, predicted in previous real space experiments [1]. We have also described previously that, using magnetic levitation as a microgravity simulation facility, different biological developmental stages have a different microgravity signature in terms of overall gene expression profile [17]. In the present article, it is shown that different ground based facilities have different effects, in terms of the number and type of genes affected. The effects are related to environmental conditions that are nearly unavoidable during space trips: flies exposed to altered gravity levels during metamorphosis are affected by simulated altered gravity especially under suboptimal growth conditions. These results are consistent with those of previous experiments in real microgravity conditions [1]; that the transcriptome is finely tuned to Earth’s gravity and the synergic effects exerted by altered gravity and environmental suboptimal conditions may be explained by a “out of tune” state of the transcriptome in these ecological conditions, which have never before been experienced by life during its evolution.

## Competing interests

The authors declare that they have no competing interests.

## Authors’ contributions

RH carried out all experiments, both RPM and magnet sample processing, molecular genetic studies and data analyses and drafted the manuscript. ILV performed RNA amplification and Affymetrix microarray hybridizations and preliminary data analysis. OJL, RJAH (magnet system) and JJWAL (RPM/LDC) developed the experimental apparatus, performed the calculations of the magnetic field and effective gravity, operated and advised on the use and effects of each simulation system. FJM participated in the study design and coordination and helped to draft the manuscript. All authors read and approved the final manuscript.

## Supplementary Material

Additional file 1 **Supplementary online material containing affected gene lists used in DAVID analysis (from Table** 2 row 1). Click here for file

Additional file 2 Supplementary online material containing seven DAVID GO cluster figures. Click here for file

Additional file 3 Supplementary online material containing the microarray signal log2ratios for each comparison. Click here for file

Additional file 4 Supplementary online material containing the GEDI analysis source files. Click here for file
